# Supplementary figures and images for: Distinct Whole Transcriptomic Profiles of the Bursa of Fabricius in Muscovy Ducklings Infected by Novel Duck Reovirus with Different Virulence
Source: Viruses. 2022 Dec 30;15(1):111. doi: 10.3390/v15010111 (PMC9866435; doi:10.3390/v15010111)

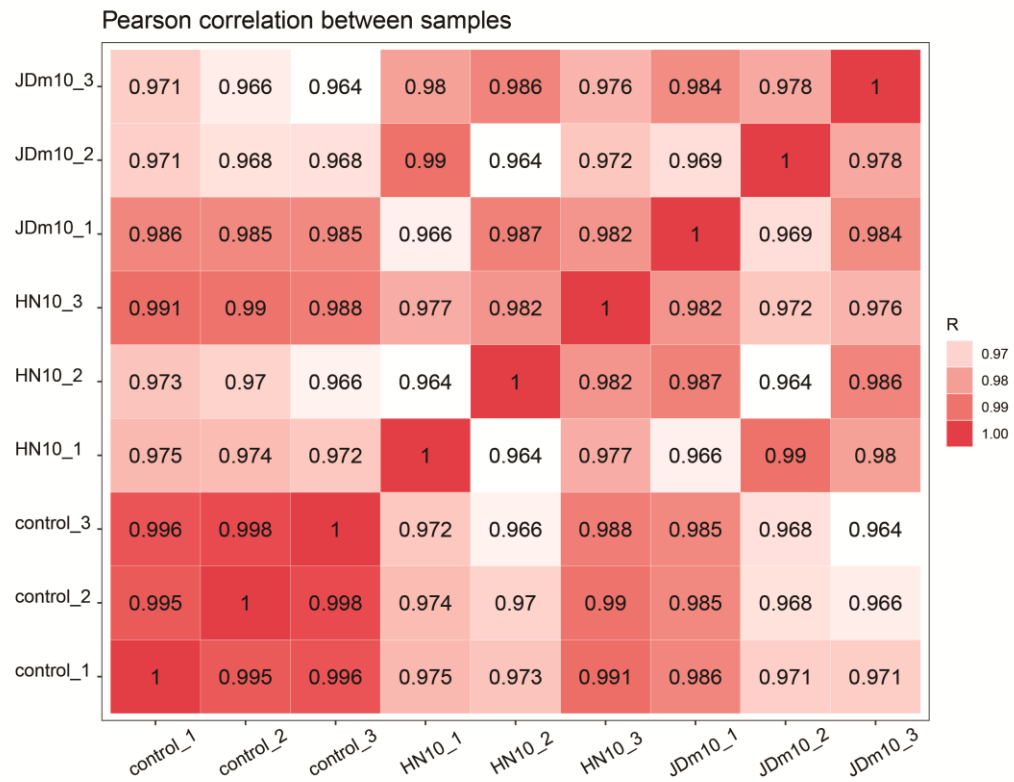

Figure S1: Pearson correlation among the duplicate samples of each group of RNA-seq

Supplement: Supplementary file 1 [file viruses-15-00111-s001.zip › Figure S1.pdf]
